# Higher EU-TIRADS-Score Correlated with BRAF V600E Positivity in the Early Stage of Papillary Thyroid Carcinoma

**DOI:** 10.3390/jcm10112304

**Published:** 2021-05-25

**Authors:** Karolina Skubisz, Joanna Januszkiewicz-Caulier, Patrycja Cybula, Elwira Bakuła-Zalewska, Krzysztof Goryca, Agnieszka Paziewska, Filip Ambrożkiewicz, Kosma Woliński, Michał Mikula, Jerzy Ostrowski, Marek Dedecjus

**Affiliations:** 1 Department of Gastroenterology, Hepatology and Clinical Oncology, Centre of Postgraduate Medical Education, 02−781 Warsaw, Poland; karolina.skubisz@gmail.com (K.S.); patrycjacybula@interia.pl (P.C.); agapaziewska@poczta.onet.pl (A.P.); jostrow@warman.com.pl (J.O.); 2 Department of Oncological Endocrinology and Nuclear Medicine, Maria Sklodowska-Curie National Research Institute of Oncology, 02-781 Warsaw, Poland; j.januszkiewicz@poczta.fm; 3 Department of Pathology, Maria Sklodowska-Curie National Research Institute of Oncology, 02-781 Warsaw, Poland; elwirabz@onet.pl; 4 Next-Generation Sequencing Core Facility, Centre of New Technologies UW, 02-097 Warsaw, Poland; kgoryca@gmail.com; 5 Department of Genetics, Maria Sklodowska-Curie National Research Institute of Oncology, 02-781 Warsaw, Poland; filipambrozkiewicz@gmail.com (F.A.); michal.mikula@pib-nio.pl (M.M.); 6 Department of Endocrinology, Metabolism and Internal Medicine, Poznan University of Medical Sciences, 60-356 Poznań, Poland; kosma1664@poczta.onet.pl

**Keywords:** BRAF V600E, papillary thyroid carcinoma, EU-TIRADS, histological aggressiveness, overtreatment

## Abstract

The data demonstrating a correlation between sonographic markers of malignancy of thyroid cancer (TC) and its genetic status are scarce. This study aimed to assess whether the addition of genetic analysis at the preoperative step of TC patients’ stratification could aid their clinical management. The material consisted of formalin-fixed paraffin-embedded tumor fragments of 49 patients who underwent thyroidectomy during the early stages of papillary TC (PTC). Tumor DNA and RNA were subjected to next-generation sequencing (NGS) on Ion Proton using the Oncomine™ Comprehensive Assay panel. We observed a significant correlation between BRAF V600E and a higher EU-TIRADS score (p-value = 0.02) with a correlation between hypoechogenicity and taller-than-wide tumor shape in analysed patients. There were no other significant associations between the identified genetic variants and other clinicopathological features. For TC patient’s stratification, a strong suspicion of BRAF V600E negativity in preoperative management of TC patients could limit the over-treatment of asymptomatic, very low-risk, indolent disease and leave room for active surveillance.

## 1. Introduction

Despite considerable progress in the comprehension of thyroid carcinogenesis, the assessment and stratification of thyroid cancer (TC) patients are still controversial. Broad access to high-quality thyroid ultrasonography (US) and fine-needle aspiration biopsy (FNAB) allows the detection of small, subclinical TCs and results in rapid treatment and an excellent survival rate. On the other hand, a huge group of patients with indolent and low mortality disease are over-treated. A rapidly growing number of TC cases (accounting for 3.8% of all cancers diagnosed annually worldwide and placing itfifth among the most common cancers in women) [1] in addition to TC’s economic impact, which cannot be disregarded, make the proper assessment of TCs one of the major concerns of endocrine oncology [2,3]. TC is one of the most heterogenic malignancies ranging from thyroid microcarcinoma to anaplastic TC, which has particularly poor outcomes. For papillary thyroid carcinoma (PTC),which relatesto 70–80% of all TCs, 16 histological variants are described [4], 5 of which are associated with aggressive cancer behavior. It can be expected that different histological types result from different molecular landscapes and should have an impact on the sonographic features of TC. However, data demonstrating a clear association between both are scarce and usually contradictory.

BRAF mutation, which is present in 40–65% of PTC cases and predominantly p. V600E (found in 45% of PTCs) represents the genetic marker of this carcinoma. An association between BRAF mutation positivity and aggressive tumor phenotype (extrathyroidal extension, lymph node metastasis) hes been reported, with a higher risk of recurrent and persistent disease [5,6], especially when TERT promoter mutation coexists [7,8]. Moreover, a linear association between TC mortality and the age of patients with BRAF V600E mutations has been observed (independent of other clinicopathologic risk factors) [9]. Retrospective multicenter studies have demonstrated that the presence of BRAF V600E mutation is significantly associated with poorer PTC outcome [6] and increased cancer-related mortality among patients with PTC [10]. Other data associates BRAF V600E with reduced follicular cell differentiation and lower iodine uptake and metabolism [11]. Some authors claim age and male sex to be strong, continuous, and independent mortality risk factors in patients with BRAF V600E mutation, but not in patients with wild-type BRAF [9,12], others do not report a negative prognostic impact of BRAF V600E mutation status on survival [13], or for aggressive tumor behavior in conventional and follicular variants of PTC [14,15]. The same discussion persists as to how the genetic profile of TC is translated to its US aspect.US features of high risk of malignancy, according to EU-TIRADS classification, such as blurred margins and microcalcifications were demonstrated to be independent predictors of BRAF V600E presence [12]. Moreover, associations with other sonographically-alerting findings, such as marked hypoechogenicity [16], solid structure, taller-than-wide shape, and absence of halo [17] microcalcifications and mixed-type non-increased vascularity [18] were also demonstrated in BRAF V600E positive carcinomas. Although other studies contradict the aforementioned findings [19,20] BRAF V600E may not only predict a histopathologic diagnosis of PTC, but also serve as a marker for more aggressive cancer phenotypes.

In the current study, we analysed the genetic profile of PTC in early clinical stages to define the genotype of more aggressive thyroid cancer phenotypes. In addition to the use of a standard stratification algorithm including a US/FNAB combination at the pre-surgery step and histologic evaluation post-operatively, 161 oncogenesis associated genes were sequenced and analysed for the presence of mutations. We aimed to assess, at the preoperative step of TC patients’ stratification, whether the addition of genetic analysis to standard management could help to differentiate the group of patients who will potentially benefit from a less aggressive approach.

## 2. Materials and Methods

### 2.1. Patients

A total of 49 patients (41 females, 8 males, median age 45 years, range 20–80 years) who underwent thyroidectomy for different types and stages of PTC at the Maria Sklodowska-Curie National Research Institute of Oncology, Warsaw, Poland between 2015 and 2016 were eligible for this study. A standard preoperative diagnostic procedure was applied, including thyroid US (scored according to EU-TIRADS) followed by FNAB, described with the Bethesda System for Reporting Thyroid Cytopathology (BSRTC). In all tumors, post-operative histopathological verification was performed allowing for PTC subtype classification. This retrospective study was approved by the institutional bioethical review board of the Maria Sklodowska-Curie National Research Institute of Oncology in Warsaw, Poland.

### 2.2. DNA and RNA Extraction

Formalin-fixed, paraffin-embedded (FFPE) tissue samples were macrodissected from tumor tissue to obtain 100% tumor cells for nucleic acids isolation. DNA and RNA were isolated from tumor cells’ FFPE samples using the RecoverAll™ Total Nucleic Acid Isolation Kit (Thermo Fisher Scientific, Waltham, MA, USA) according to the manufacturer’s protocol. DNA and RNA quality and quantity were assessed using a NanoDrop spectrophotometer and Qubit fluorometer. DNA was stored at −20 °C and RNA at −70 °C while awaiting further analysis.

### 2.3. Libraries Preparation and Sequencing

Before the library construction, a reverse transcription process was conducted to obtain cDNA from RNA. DNA and cDNA libraries were prepared using Oncomine™ Comprehensive Assay v3 (Thermo Fisher Scientific, Waltham, MA, USA), according to the manufacturer’s protocol. The panel covers 161 cancer driver genes, including kinase domain and DNA repair genes. The libraries concentrations were analysed using an Agilent 2100 Bioanalyzer. Sequencing was performed using the Ion Proton instrument with the Ion PI Hi-Q Sequencing 200 Kit (Thermo Fisher Scientific, Waltham, MA, USA).

### 2.4. Data Analysis

Variants were named using Ion Reporter (version 5.10), using “Oncomine Comprehensive v3—w3.2—DNA and Fusions—Single Sample” protocol. Variant calling parameters were as follows: Fusions panel: “v3 Fusions v1.2”; target regions: “v3 Regions v1.1”; hotspots: “v3 Hotspots v1.1”; annotations: “v3 Annotations v1.2”; reference genome:hg19... Multiple variants called for a single locus were split using vcfbreakmulti tool from vcflib package (https://github.com/vcflib/vcflib) (accessed on 3 May 2018). Variants were trimmed and aligned to the leftmost position with the Genome Analysis Tool Kit (version 3.8.0, [21]). Variants were filtered with bcftools (version 1.3) using the following parameters for all variants: minimum sequencing depth (DP): 20, minimum quality (QUAL): 20, genotype quality (GQ) greater than 5. Additionally, for single/multiple nucleotide polymorphisms the following filters were applied: flow evaluator read depth at the locus (FDP) > 6, flow evaluator alternate allele observations (FAO) > 2, strand bias in variant relative to the reference (STB) < 0.9. For indels the following additional filters were applied: FDP > 10, FAO > 4, and the number of consecutive repeats of the alternate allele in the reference genome (HRUN) < 6. Filtered variants were annotated using Annovar [22].

Annotated variants were loaded into R (version 3.4.1), where these additional filters were applied: at least 2 alternate reads from each strand, alternate reads fraction greater than 0.1. Variants present in more than 0.1% of the population according to the 1000 Genomes Project database, the Exome Sequencing Project of the National Heart, Lung, and Blood Institute (6500 exomes, [23]) and in the Exome Aggregation Consortium database (ExAC, > 60,000 exomes, [24]) were removed.

### 2.5. The Cancer Genome Atlas (TCGA) PTC Dataset Exploration

Genomic and clinical data of 496 patients (342 females, 125 males, median age 46 years, age range 15–89) with diagnosed papillary thyroid cancer, were obtained from TCGA (The Cancer Genome Atlas) and analyzed using cBioPortal [25].

## 3. Results

Forty-nine FFPE samples extracted from patients with papillary thyroid cancer were sequenced using Ion Proton. Among these 21 (43%) samples were classical variants, 16 (33%) were follicular variant, 8 (16%) were oxyphilic variant, and 4 (8%) were diffuse sclerosing variants.

**Figure 1:**
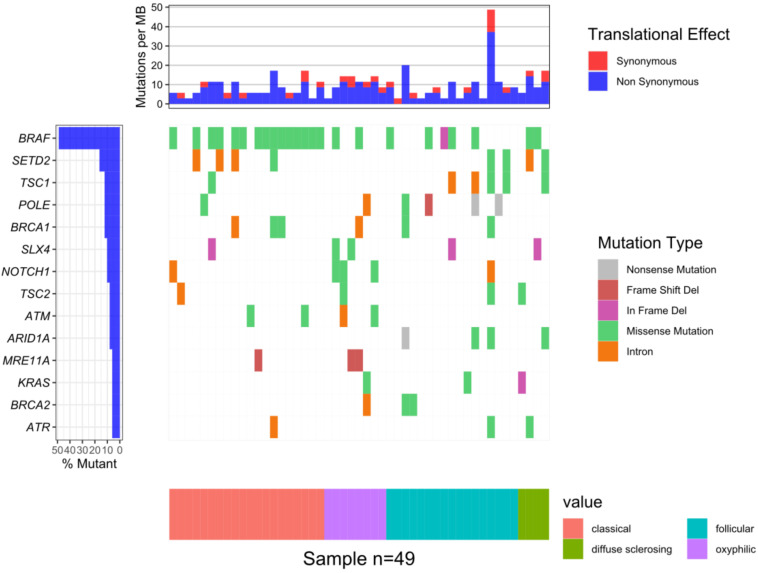
The waterfall plot of 14 genes in which mutations were identified in at least of 5% patients: BRAF—24 samples (49%); SETD2—8 (16%); TSC1, POLE, BRCA1—6 (12%); SLX4, NOTCH1—5 (10%); TSC2, ATM, ARID1A—4 (8%); MRE11A, KRAS, BRCA2, ATR—3 (6%).

The median of the mean coverage per sample was 2856 and the median for the percent of bases with coverage more than 100× was 99.5%. A total of 130 somatic mutations were identified in 57 genes out of 161 present in the Oncomine™ Comprehensive Assay v3 (Supplementary Material Table S1). The highest number of mutations was observed in the BRAF gene, specifically, the V600E variant was observed at a frequency of 74% (14/19), 33% (3/9), 24% (4/17) and 50% (2/4) in the classical, oxyphilic, follicular and diffuse sclerosing types, respectively. In five patients gene fusions of EIF3E-RSPO2, SND1-BRAF, CCDC6-RET and ETV6-NTRK3 were detected (Supplementary Material Table S1). The most common mutations identified are presented in Figure 1.

After the exclusion of five patients for data ambiguity (data missing, unclear assignment of PTC subtype) we performed a final analysis on 44 PTC samples. The incidence of BRAF V600E mutation was significantly more frequent (*p*. value < 0.05) in a classical PTC subtype. There were no other significant associations between identified genetic variants and other clinicopathological features. Next, the BRAF V600E status was compared to main US features. The main characteristics of BRAFV600E-positive and BRAF-V600E- negative patients’ groups are presented in Table 1.

**Figure 2:**
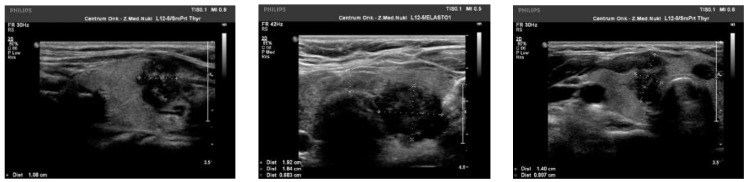
US image of BRAF V600E positive PTCs: solid structure (2 points), deeply hypoechogenic, (3 points), taller-than-wide orientation (3 points), blurred margins (2 points), microcalcifications (3 points)—13 points = TIRADS 5.

**Figure 3:**
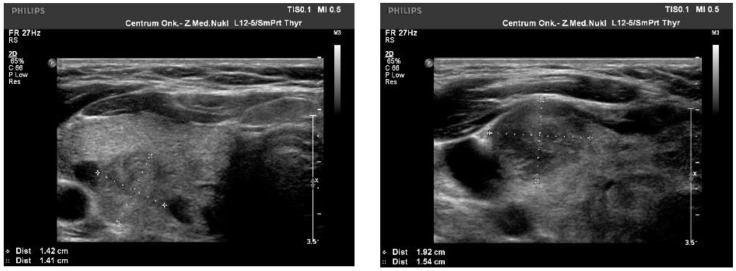
US image of BRAF V600E negative PTCs: solid structure (2 points), isoechogenic or slightly hypoechoic (1–2 point), taller-than-wide orientation (3 points), blurred margins (2 points), absence of calcifications (0 points)—8–9 points = TIRADS 5.

We observed the strongest correlation between hypoechogenicity (*p*-value < 0.05) and BRAF V600E positivity, although four more malignancy-associated features (taller-than-wide shape, blurred margins, microcalcifications, and absence of “halo”) were also more frequent in the BRAF V600E (+) group. PTC risk increased with a higher EU-TIRADS score (*p*-value = 0.04). US images of BRAF V600E (+) and BRAF V600E (−) PTCs are presented in Figure 2 and Figure 3, respectively.

No statistically significant difference was observed, as expected, between BRAF V600E status and FNAB results. There were no important differences between features representing TNM staging (maximal diameter, multifocality, angioinvasion lymph node metastasis at diagnosis) and BRAF V600E status except for capsule invasion or excision which, for BRAF V600E (+),was almost twice as often than for BRAF V600E (–) and multifocality was more than twice as often as BRAF V600E (–);however, the difference did not reach the level of statistical significance (*p*-value = 0.08) (Table 1). Additionally, for one of the diagnosed patients (female, age 48) SND1-BRAF fusion was identified (2.3% of all samples).

According to data from exome and whole genome sequencing of 496 papillary thyroid carcinomas from the TCGA project, BRAF somatic mutations were identified in 246 (62%) of 399analysed patients. A total of 96% of them were V600(+) missense mutations. BRAF fusions were identified in 1.5% of all samples, with SND1-BRAF fusions mainly discovered [25,26].

## 4. Discussion

According to the current literature review BRAF V600E (+) PTC subtypes demonstrate various US landscape. A papillary or a mixed growth pattern of the BRAF V600E(+) oncocytic variant of PTC has been was described to be hypo- or isoechogenic with smooth or spiculated/microlobulated margins, a non-parallel orientation and mixed vascularity (increased in half of the cases), with an absence of microcalcifications and macrocalcifications found in 20% of tumors [20]. According to the available literature data, the ultrasonographic characteristics of the follicular PTC variant (FVPTCs) are generally less suspect than those of the classical variant with US features such as taller-than-wide shape, microcalcifications, marked hypoechogenicity and blurred margins being less frequent in FVPTCs, especially when the tumor size exceeds 1 cm [27]. Furthermore, patients with FVPTCs which presented with malignant US features have a worse prognosis [28]. Zhang et al., propose broadening the FNAB criteria when the nodule has FVPTC US features [29]. On the other hand, Kim et al., demonstrated no statistical significance for ultrasonographic high-suspicion features in BRAF V600E mutation-positive vs BRAF V600E (–) FVPTC patients, although FVPTC BRAF V600E (–) patients showed more frequent encapsulation and halo signs than BRAF V600E (+)patients [30]. Due to limited studies, a clear association between BRAF V600E mutation status and poor clinicopathological features in FVPTC has not yetbeen established. The tall cell PTC variant, (BRAF V600E (+) in over 92%) has been described as a solid, hypoechogenic tumor with a spiculated/microlobulated margin, and a non-parallel orientation and with frequent nodal metastases [31]. The columnar cell variant (BRAF V600E (+) in over 30%) features larger hypoechoic nodules, with microlobulated margins, which are often associated with visible extrathyroidal invasion and lymph node metastases [31]. The hobnail variant (BRAF V600E (+) in almost 60%) is usually microlobulated and hypoechoic, with microcalcifications and multiple lymph node metastases reported [31]. The BRAF V600E (+) Warthin-like variant (WV), frequently associated with Hashimoto’s thyroiditis (HT) (93% to 100% of all cases) [31] presents with US features of malignancy, suchas solid composition, hypoechogenicity, and taller-than-wide shape [32]. BRAF V600E (+) nodules in HT patients have a rather solid structure, while BRAF V600E (+) nodules in patients without HT have microcalcifications [33].

The clinical utility of BRAF V600E screening has been a subject of various studies, but—to date, no consensus has been reached. It has been demonstrated that BRAF V600E mutation analysis can improve the diagnostic performances of FNAB [34,35] especially in the case of indeterminate cytology according to BSRTC [36,37,38]. In this “grey zone” of FNAB results, the expected risk of cancer is 5–15%, 20–30%, and 50–75%, for categories three (atypia or follicular lesions of undetermined significance), four (follicular neoplasms or suspicious for a follicular neoplasm) and five (suspicious for malignancy), respectively [39,40]. Such uncertainty might cause a dilemma to both patients and clinicians and often leads to over-diagnosis and over-treatment, affecting the patient’s quality of life, financial costs, and cancer stigmatisation [41].

No agreement has been reached as to the additional value of BRAF V600E status as a screening tool. Despite a high specificity for thyroid cancer, BRAF V600E mutation has a low overall sensitivity and therefore has a limited diagnostic value as a single screening test [42,43]. According to some authors, the benefit of BRAF V600E mutation testing in surgical planning is unclear and mutation status may not add additional insight compared with routine preoperative neck US [44]. On the other hand, although BRAF V600E mutation has been more frequently found insuspicious nodules on US than in those with a benign US aspect [45,46], it has been reported that BRAF mutation analysis brings additional diagnostic value in thyroid nodules with the“suspicious for malignant” cytology alone, even when the nodules do not present alerting US features [47]. Hahn et al., found that hypoechogenicity and a nonparallel orientation were associated with BRAF mutation positivity [48]. Conversely, other studies deny the close correlation between suspicious US features and BRAF mutation [49,50,51]. The sensitivity of BRAF V600E mutation analysis has been proven to be so high for PTC, that even if benign cytology with positive BRAF V600E mutation were reported, more aggressive management should still be considered [52]. Consequently, some authors recommend, that fine-needle aspiration should be routinely accompanied by the BRAF V600E mutation test in high-risk thyroid nodules with ≥2 suspicious ultrasound features [53]. As postulated by Zhang et al. [37], EU-TIRADS could be used as the preliminary evaluation method to select high-risk lesions for FNAB, while BSRTC and BRAF V600E mutation analysis should be adopted to refine the diagnosis, as an increased level of EU-TIRADS classification was significantly associated with the rising mutation rate of BRAF V600E in each BSRTC category.

Postoperative use of BRAF V600E mutation testing to guide complementary radioactive iodine treatment is also a subject of discussion [54]. The BRAF V600E mutation has been found to not be associated with an incomplete response during follow-up, despite its correlation with older age and advanced tumor stage [55]. In some studies, it represents the worst outcome for PTC patients, independently of other clinicopathological features [56], while more recent studies show no such correlation [57]. The most modern techniques such as contrast-enhanced US, also demonstrate that BRAF mutation-positive nodules usually have a larger size, hypo-enhancement, centripetal enhancement, inhomogeneous enhancement, complete enhancement, blurred boundaries, an irregular shape, and a washout period at preoperative CEUS than those without BRAF mutations [58,59].

As we have observed an obvious data noise relating to modern, individually tailored TC treatment we aimed to assess whether the addition of molecular analysis could narrow the group of patients who would benefit from less aggressive treatment at the preoperative stratification step. Although a relatively small group of patients was taken into consideration, the BRAF V600E positivity clearly increased with a higher EU-TIRADS score, with the strongest correlation found between hypoechogenicity and taller-than-wide tumor shape. The correlation between the presence of more aggressive histological findings (capsule invasion or excision) is in full coherence with the above-mentioned status of knowledge regarding BRAF V600E mutation. Our aim was, however, to suggest a perceptive waiting strategy for patients without an elevated EU-TIRADS score on the preoperative US especially in cases of small (<1 cm) indolent tumors, before they are stigmatised with cancer suspicion.

Our study has several limitations. First, the possibility of selection bias exists as this was a retrospective study from a single institution. Secondly, reliable results were difficult to achieve because of the small datasets, and we will aim to continue our observations on more numerous medical records. Additionally, preoperative US was performed with the same US machine set with similar parameters to avoid equipment-based variability, but by different clinicians which may introduce the possibility of subjectivism. Thirdly, we focused on the role of the BRAF status of patients with PTC, although a vast oncogenesis-associated gene panel was analysed (exact genetic results not yet published). Most patients with PTC have a favorable prognosis after surgery. They are more likely to die from other diseases, although it is known that recurrence and death can occur more than 30 years after the initial diagnosis of PTC [59].The American Thyroid Association guidelines up-dated and published in January 2016 recommended a ”less is more” approach for the management of well-differentiated TC [54];-, however the aplication of this advice in clinical practice is not yet common, as its interpretation is often subjective, leaving place for different interpretation in different clinical centers [57]. Both a personalised approach and risk assessment are recommended in each case, with numerous possible options: active surveillance, lobectomy, or total thyroidectomy with or without adjuvant radioactive iodine treatment. As some high-risk characteristics are determined only after the final pathology results, some patients who undergo initial thyroid lobectomy may require completion thyroidectomy as a second procedure [60].

Taking the results presented in this study into consideration, we suggest that the stratification of patients in the early stages of PTC needs to be revised and more individually-tailored. Even a single gene mutation analysis which is not a highly expensive procedure (and even more, probably does not require an additional FNAB [58]), in cases of US “worrisome” nodules could alter a patient’s management. In an era of easily available invasive treatments (lobectomy or thyroidectomy), which are most frequently proposed by physicians and demanded by patients when a cancer diagnosis is assessed, we observe an increasing number of overtreated patients having to bear the consequences of adverse effects sometimes for the rest of their lives. Therefore we propose the addition of BRAF V600E analysis for high-TIRADS scored PTCs, as it as a reliable, cost-effective tool which potentially assists a group facing the most probable aggressive PTC outcome. Such patients could benefit from the classical surgery-radioiodine treatment path. On the other hand, low EU-TIRADS -scored US images of PTC seem to be a weaker candidate for BRAF V600E mutation positivity; however, additional evaluation of the negative BRAF V600E status for early PTCs could serve as an additional argument for active observation rather than classical management in such patients.

## 5. Conclusions

Our results need further validation in a larger dataset to better estimate their potential clinical use; however, in addition to the growing consensus, we would like to emphasize the fact that we already dispose of reliable diagnostic tools to differentiate between more aggressive PTCs and tumors with most probable indolent phenotypes in the preoperative step of patient’s stratification. Not one of them alone is sufficient to properly stratify a patient’s risk. Analysis of a multigene panel covering over 160 cancer driver genes is an expensive diagnostic tool and we are aware that the use of this technique may be not easily feasible for less affluent medical centers. However, a complement of BRAF status seems to be a very reasonable and cost-effective approach in a case involving an aggressive PTC phenotype suspicion, based on the combination of numerous “standard” diagnostic methods (including clinical, cytological, and ultrasound suspicious features). An estimation of BRAF V600E status in the initial course of the disease could not only help to avoid overtreatment in a patient with a less aggressive cancer phenotype (BRAF negative PTCs) but also to predict the treatment response in those with a BRAF positive status. This would allowfor the selection of patients who could potentially benefit from oncogene-targeted therapy rather than from e routine post-operative additive therapy.

## Figures and Tables

**Table 1:** Main characteristics of BRAF V600E positive and BRAF V600E negative patients’ groups.

	BRAF(+), N = 21 Cs	BRAF(-), N = 23 Cs	*p*-value
Demographic characteristics			
Age	45.1, SD 16.1	45.6, SD 13.7	0.91
Male/Female ratio	3 M,18 F	5 M, 18 F	0.52
Histopathological characteristics			
Max diameter [mm]	12.7, SD 6.1	15.3, SD 12.5	0.38
Multifocality	42.9%	21.7%	0.13
Angioinvasion	42.9%	34.8%	0.58
Capsule infiltration or extrathyroidal extension	47.6%	21.7%	0.07
Lymph node metastases at diagnosis	42.9%	34.8%	0.58
Sonographic characteristics (pre-surgically)			
Solid	94.7%	95.5%	0.92
Hypoechogenic	100,0%	77.3%	0.03
Taller-than-wide	68.4%	40.9%	0.08
Irregular margins	95.2%	78.3%	0.10
Microcalcifications	52.4%	43.5%	0.55
Absence of “halo”	81.0%	60.9%	0.14
EU-TIRADS, points	10*	8.5*	0.04

## Data Availability

Public data availability is not allowed. Data was recovered from the institutional medical files.

## Supplementary Materials

The following are available online at https://www.mdpi.com/article/10.3390/jcm10112304/s1, Table S1: List of variants detected in assessed clinical samples. Gene–gene name; ExonicFunc/gene fusion isoform–consequence of variant/gene fusion isoform identifier CHROM/POS-genome coordinates of a variant; REF/ALT-reference/alternative variant sequence; AAChange–Amino acid change; esp6500siv2_all-variant frequency according to National Heart, Lung, and Blood Institute GO Exome Sequencing Project; SIFT/Polyphen2-prediction of variant impact on protein structure: B-benign, T-tolerated, D-deleterious; ClinVar–ClinVar clinical significance
